# Socio-demographic and behavioural characteristics of illegal motorcycle street racers in Malaysia

**DOI:** 10.1186/1471-2458-11-446

**Published:** 2011-06-08

**Authors:** Li Ping Wong

**Affiliations:** 1 Department of Social and Preventive Medicine, Faculty of Medicine, University of Malaya, 50603, Kuala Lumpur, Malaysia; 2 Julius Centre University of Malaya (JCUM), Faculty of Medicine, University of Malaya, Kuala Lumpur, Malaysia; 3 Centre of Population Health (CePH), University of Malaya, Kuala Lumpur, Malaysia

## Abstract

**Background:**

This study sought to understand the factors associated with street racing among the illegal motorcycle racers in Malaysia or known as the *"Mat Rempit"*.

**Methods:**

Street outreach interviewer-administered surveys were conducted from June 2008 to January 2009 in this multi-state study.

**Results:**

A total of 2022 participants were surveyed, the mean ± *SD *age of the participants was 20.5 ± 3.4 years (age range: 12 to 35 years). Mean duration of street racing was 2.65(*SD *± 1.77) years (range: 2 months to 12 years), with 50.1% and 35.8% reporting stunt riding and alcohol drinking while racing, respectively. With regard to risk behaviours, cigarette smoking was highly prevalent among the study participants (78.3%), followed by alcohol drinking (27.8%) and recreational drug use (18.8%). Participants scored high on the masculinity scale (15.7 ± 4.0 out of 21.0). The results of the logistic regression analysis showed that socio-demographic variables, risk behaviour and masculinity scores were associated with racing frequency.

**Conclusion:**

Given these associations, tailoring family-centered interventions to the needs of the lower socio-economic groups and interventions recognizing the negative consequences of health risk behaviours related to street racing as an expression of traditional masculinity should be emphasized.

## Background

Street racing has long been identified as a threat to civil society with significant social and economic impact [1]. Street racing threatens the lives of other road users and causes unnecessary nuisance to the public. In Malaysia, there is a strong motorcycle racing culture among some Malaysian youth, known as "Mat Rempit" in Malay Language. Most of the Mat Rempits race with small capacity motorcycles and without proper safety measures. Most of their motorcycles are modified for higher speed or to increase the noise emitted by the exhaust, which do not meet standard specifications. The Mat Rempits usually travel in large groups and racings are usually conducted on public roads where it endangers other road users. In addition to racing, they also weave through moving traffic and perform dangerous motorcycle stunts. They are famous for their "Superman" stunts and other feats performed on their motorcycles. They are also notorious for their "Cilok" or weaving in-between moving traffic at high-speed.

Empirical research on illegal street racing is limited despite its renowned negative consequences and long existence [1]. There are numerous conceptual frameworks for understanding risk behaviours of adolescents and young adults. **A**dolescent risk-taking behaviours have been associated with a variety of demographic, parental, social and environmental factors. Studies showed that family socio-economic status and family structure may influence adolescent risk-taking behaviours [2,3]. Low household socio-economic status, measured by family income, was reported to be associated with adolescent substance use [2,4]. Family structure such as marital disruption has also been associated with cognitive, emotional and behavioural problems [3,5]. Permissive parenting style has been found to be associated with higher frequency of substance abuse and school misconduct [6]. In contrast, parental support, monitoring and strict rules and attitudes about drinking and illicit drug use were related to less adolescent substance use [7]. While there is increasing recognition that adolescent problematic behaviours are associated with family-related variables, research examining problem behaviours such as motorcycle street racing remains limited. Studies investigating car racing have shown that most of those involved were from a lower to middle class background [8-10].

Jessor and Jessor's Problem Behavior Theory proposes that problem behaviours manifest in a variety of interrelated deviant, norm-violating, or health-compromising behaviours and reflect a basic underlying propensity [11,12]. There were some well-publicized empirical studies that showed adolescent and early adult problem behaviour tends to co-occur with other risky or problematic behaviours such as excessive alcohol consumption, substance abuse and sexual activity [13,14]. In particular, risky driving behaviours were also shown to be associated with cigarette smoking, binge drinking and drug use [15,16]. However, studies examining the association between risky or problematic behaviours and illegal motorcycle street racing have never been conducted and warrant further investigation.

Masculinity ideology has been shown to be related to health attitudes and problematic behaviours. For example, men with traditional masculine beliefs are less likely to report practicing health protective behaviours [17], and show greater substance use, including smoking, excessive alcohol consumption, and high-risk sexual activity [18-20]. The association between car racing and masculinity has also been hypothesized [1,20-22]. Research on masculinity has suggested that respect, honour, courage, aggressiveness, dominance, toughness, and the willingness to resort to violence to resolve interpersonal conflicts are central characteristics of masculine identity [20]. A causal link between masculinity and speeding has also been demonstrated [21]. Illegal car street racing has been regarded as is a symbol of masculine power, socially constructed as the epitome of masculinity, and a form of manifestation of the norms of masculine culture [8,20]. It has also been suggested that men strive to express their masculinity through risky activity such as reckless driving [22]. Additionally, our recent qualitative investigation on a subsample of illegal motorcycle street racers (Wong LP: In-depth understanding of health risk behaviors and needs of illegal motorcycle racers in Malaysia, submitted) as well as other studies [9,10] also revealed that respect- and honor-seeking for being brave were among the identified characteristics of masculine identity associated with street racing. Considering the important theoretical and practical implication of masculinity in this context, sound research evidence is warranted in the unique phenomena of the illegal motorcycle racing in Malaysia.

No empirical study to date has assessed the problem behaviours of illegal street racers in Malaysia. This study sought to investigate socio-demographic and behaviour characteristics of illegal motorcycle racers. The behaviour characteristics investigated included racing activities, health behaviour, and self-rated level of masculinity. It is hoped that the findings from this study may provide important insights that will inform the development of behavioural intervention programs to reduce adolescent and young adult risk-taking behaviours associated with illegal motor racing.

## Methods

The study employed a snowball sampling technique (chain referral sampling method that relies on referrals from initial participants to generate additional participants), an established method to sample hard-to-reach populations because of moral, legal social sensitivities surrounding the behaviour under investigation [23]. Due to the difficulty in gaining access to the population of illegal motorcycle racers, all the interviews were conducted by trained interviewers selected among the illegal motorcycle racers, in an attempt to facilitate access to the target groups and settings. Inclusion criteria were as follows: (1) self-identified as illegal motorcycle street racer or "*Mat Rempit*"; and (2) engage in illegal motorcycle street racing at the time of interview. Sample size was not predetermined because the exact number of illegal motorcycle racers in the country was unknown. Participants were recruited using street outreach approach throughout all the states in Malaysia. Data collection was carried out from 22 June 2008 to 4 January 2009. All participants were informed of the study's objectives and signed an informed consent form. They were assured that their responses would be confidential and reminded that their participation in the study was voluntary. The study was approved by the Medical Ethics Committee, University Malaya Medical Center, Kuala Lumpur, Malaysia.

All consenting participants were interviewed using a standard questionnaire prepared to gather demographic characteristics, illegal racing behaviour, health behaviour, and self-perceived masculinity. These outcomes were investigated on the basis of past research which indicated their associations with youth problem behaviours [6,18,24-26]. The first section of the questionnaire included questions on participants' background information and family characteristics. The second section consisted of 5 questions designed to gauge involvement in illegal motor racing. The third section investigated participants' health behaviour through an 8-item health protective behaviour scale (diet and health care). Each response was coded either yes (1) or no (0). Scale item responses were scored in the direction of high protective behaviour. Thus, higher scores on the scale are indicative of higher health protective behaviour. This scale had moderate internal consistency (Cronbach's alpha = 0·668). The 5-item scale measuring health risk behaviour consisted of questions measuring substance abuse (smoking, alcohol drinking and drug use) and sexual risk (multiple sexual partner and safe sex practices). Item responses are scored either yes (1) or no (0). Thus, in contrast to health protective behaviour score, a higher health risk behaviour score is indicative of high risk behaviour. The health risk behaviour scale had moderate internal consistency (Cronbach's alpha = 0.624). The last section measured participants' self-rated levels of masculinity. The masculinity score is the mean of the ratings for a total of 7 items (strong personality, courage, assertive, respect, willing to take risks, dominant). Participants were asked to respond either *Strongly Disagree, Disagree, Agree *or *Strongly Agree*. Responses to this item were scored from 0 to 3 in the direction of increasing masculinity. The internal consistency of the masculinity scale was high (Cronbach's alpha = 0.869). The questionnaire was pilot tested prior to administration.

### Analyses

Data was analyzed using SPSS 17.0 for Windows. The score distributions were checked for underlying assumptions of normality using the Kolmogorov-Smirnov test; as all the score distributions were not normally distributed non-parametric tests were used. The Mann-Whitney *U *test and the Kruskal-Wallis test were used for comparison of medians. Spearman's correlation coefficient was used for correlation analyses. The chi-square test was used to test the significance of differences in percentages. Multivariate analysis, using binary logistic regression analysis, performed using the ENTER method, was used to determine factors associated with frequency of racing and stunt riding. All significant variables (*p *< 0.05) associated with frequency of racing and stunt riding in the univariate analyses that were considered important based on the *a priori *hypotheses were included in the multivariate regression model. Odds ratios (OR), 95% confidence intervals (95% CI) and *p*-values were calculated for each independent variable. Analyses resulting in values of *p *< 0.05 were considered significant. Goodness of fit was assessed with the Hosmer-Lemeshow test, where poor fit is indicated by a significance value less than 0.05.

## Results

A total of 2022 participants from 11 states and the Federal Territory of Kuala Lumpur were interviewed during the six month study period. The response rate was high (89.0%). Reason for non-response was not because of the sensitivity of the study issue, as most of the non-respondents cited lack of time and preoccupied with other errands as the reason for not participating. The age of participants ranged between 12 and 35 years with mean age of 20.5(*SD *± 3.4) years. The majority of participants were Malays. A large number of the study participants were students and the majority of those with paid employment reported a personal monthly income less than RM1000 per month. Less than one-fifth of the participants' parents were tertiary educated. Near two-thirds reported household monthly income of less than RM2000 per month. Near three-fourths of participants have at least one authoritative parent (Table 1).

**Table 1:** Distribution of socio-demographic characteristics of participants and logistic regression analyses (N = 2022)

Socio-demographic variables	All participants N = 2022 *n *(%)	Illegal motorcycle street racing frequency n (%)			Logistic regression Model 1	Stunt riding *n *(%)			Logistic regression Model 2
		
		Very often/ Often *n *= 620	Sometimes/ Rarely *n *= 1402	*P*	Exp (B) (95.0% C.I.)	Racing and perform stunt *n *= 1014	Racing only *n *= 1008	*P*	Exp (B) (95.0% C.I.)
**Personal characteristics**									
*Ethnicity*									
Malay bumiputra	1850 (91.5)	567 (31.3)	1274 (68.9)		2.12 (1.19 - 3.76) *	938 (50.7)	912 (49.3)		2.45 (1.52 - 3.93) ***
Chinese	68 (3.4)	25 (36.8)	43 (63.2)	< 0.05	2.60 (1.20 - 5.65) *	39 (57.4)	29 (42.6)	< 0.01	2.51 (1.24 - 5.06) *
Indian	100 (4.9)	19 (19.0)	81 (81.0)		Reference	37 (37.0)	63 (63.0)		Reference
Non-Malay bumiputra^¶^	4 (0.2)	0 (0.0)	4 (100.0)			0 (0.0)	4 (100.0)		
*Age *									
15 and below	53 (2.6)	15 (28.3)	38 (71.7)			27 (50.9)	26 (49.1)		
16 to 25	1829 (90.5)	553 (30.2)	1276 (69.8)	ns	-	915 (50.0)	914 (50.0)	ns	-
26 to 35	140 (6.9)	52 (37.1)	88 (62.9)			72 (51.4)	68 (48.6)		
*Marital status*									
Never married	1926 (95.3)	583 (30.3)	1343 (69.7)			968 (50.3)	958 (49.7)		
Married	83 (4.1)	31 (37.3)	52 (62.7)	ns	-	39 (47.0)	44 (53.0)	ns	-
Separated/divorced/widowed	13 (0.6)	6 (46.2)	7 (53.8)			7 (53.8)	6 (46.2)		
*Education attainment *									
Primary school	199 (9.9)	82 (41.2)	117 (58.8)		2.09 (1.29 - 3.40) **	123 (61.8)	76 (38.2)		2.60 (1.67 - 4.04) ***
Secondary school	1454 (71.9)	462 (31.8)	992 (68.2)	< 0.001	1.87 (1.34 - 2.61) ***	767 (52.8)	687 (47.2)	< 0.001	1.99 (1.50 - 2.64) ***
University	369 (18.2)	76 (20.6)	293 (79.4)		Reference	124 (33.6)	245 (66.4)		Reference
*Occupation *									
Professional and managerial	144 (7.2)	45 (31.3)	99 (68.8)		1.59 (1.00 - 2.51) *	61 (42.4)	83 (57.6)		
Skilled workers	350 (17.3)	112 (32.0)	238 (68.0)		1.25(0.91 - 1.71)	184 (52.6)	166 (47.4)		
Unskilled workers	461 (22.8)	130 (28.2)	331 (71.9)	< 0.05	0.81 (0.59 - 1.10)	248 (53.8)	213 (46.2)	ns	-
Unemployed	299 (14.8)	99 (33.1)	200 (66.9)		1.10 (0.79 - 1.53)	152 (50.8)	147 (49.2)		
Student	768 (38.0)	234 (30.5)	534 (69.5)		Reference	369 (48.0)	399 (52.0)		
*Personal monthly income (RM) *^† ^‡									
500 and below	54 (5.9)	12 (22.2)	42 (77.8)			26 (48.1)	28 (51.9)		
501-1000	613 (66.6)	200 (32.6)	413 (67.4)	ns	-	326 (53.2)	278 (46.8)	ns	-
1001-2000	219 (23.8)	55 (25.1)	164 (74.9)			111 (50.7)	108 (49.3)		
above 2001	35 (3.8)	12 (34.3)	23 (65.7)			14 (40.0)	21 (60.0)		
*Locality*									
Urban	1114 (55.1)	348 (31.2)	766 (68.8)	ns	-	571 (51.3)	543 (48.7)	ns	-
Rural	908 (44.9)	272 (30.0)	636 (70.0)			443 (48.8)	465 (51.2)		
**Family characteristics**									
*Parental marital status*									
Married	1736 (85.9)	481 (27.7)	1255 (72.3)	< 0.001	0.47 (0.35 - 0.63)***	849 (48.9)	887 (51.1)	< 0.05	0.91 (0.68 - 1.22)
Separated or divorced	286 (14.1)	139 (48.6)	147 (51.4)		Reference	165 (57.7)	121 (42.3)		
*Household monthly income (RM) *^† ^‡									
1000 and below	385 (21.8)	136 (35.3)	249 (64.7)		2.03 (1.38 - 2.99) ***	192 (49.9)	193 (50.1)		1.02 (0.73 - 1.42)
1001-2000	710 (40.2)	231 (32.5)	479 (67.5)	< 0.001	1.84 (1.31 - 2.59) ***	373 (52.5)	337 (47.5)	< 0.05	1.20 (0.90 - 1.61)
2001-3000	363 (20.5)	105 (28.9)	258 (71.1)		1.49 (1.02 - 2.16) *	197 (54.3)	166 (45.7)		1.21 (0.87 - 1.69)
above 3001	310 (17.5)	65 (21.6)	245 (79.0)		Reference	133 (42.9)	177 (57.1)		Reference
*Maternal education attainment*									
No formal schooling	203 (10.0)	78 (38.4)	125 (61.6)		0.60 (0.33 - 1.10)	100 (49.3)	103 (50.7)		
Primary school	307 (15.2)	86 (28.0)	221 (72.0)	< 0.05	0.50 (0.31 - 0.83)**	160 (52.1)	147 (47.9)	ns	-
Secondary school	1224 (60.5)	357 (29.2)	867 (70.8)		0.78 (0.54 - 1.14)	591 (48.3)	633 (51.7)		
University	288 (14.2)	99 (34.4)	189 (65.6)		Reference	163 (56.6)	125 (43.4)		
*Paternal education attainment*									
No formal schooling	167 (8.3)	69 (41.3)	98 (58.7)		1.41 (0.76 - 2.61)	79 (47.3)	88 (52.7)		
Primary school	306 (15.1)	94 (30.7)	212 (69.3)	< 0.05	1.04 (0.65 - 1.66)	154 (50.3)	152 (49.7)	ns	-
Secondary school	1195 (59.1)	348 (29.1)	847 (70.9)		0.84 (0.59 - 1.20)	587 (49.1)	608 (50.9)		
University	354 (17.5)	109 (30.8)	245 (69.2)		Reference	194 (54.8)	160 (45.2)		
*Parental strictness*									
Neither parent strict	560 (27.7)	228 (40.7)	332 (59.3)		1.99 (1.47 - 2.68) ***	356 (63.6)	204 (39.4)		2.22 (1.68 - 2.92) ***
At least one parent strict	850 (42.1)	267 (31.4)	584 (68.6)	< 0.001	1.67 (1.26 - 2.20) ***	420 (49.4)	431 (50.6)	< 0.001	1.61 (1.26 - 2.05) ***
Both parents strict	611 (30.2)	125 (20.5)	486 (79.5)		Reference	238 (39.0)	373 (61.0)		Reference
**Health behavior**									
Health protective behavior score (mean ± SD), ranged 0-8	4.1 ± 2.1	4.2 ± 2.2	4.1 ± 2.1	ns	-	4.4 ±2.1	3.8 ± 2.1	< 0.001	1.20 (1.14 - 1.27) ***
Health risk behavior score (mean ± SD), ranged 0-5	2.1 ± 1.1	2.2 ± 1.2	1.9 ± 1.0	< 0.001	1.20 (1.08 - 1.32) ***	2.3 ± 1.2	1.8 ± 0.9	< 0.001	1.56 (1.41 - 1.72) ***
**Masculinity**									
Masculinity score (mean ± SD), ranged 0-21	15.7 ± 4.0	16.3 ± 4.2	15.4 ± 3.9	< 0.001	1.08 (1.04 - 1.11) ***	16.0 ± 4.1	15.4 ± 3.8	< 0.001	1.06 (1.03 - 1.08) ***

* *P *< 0.05; ** *P *< 0.01; *** *P *< 0.001.

† Number of respondents less than 2022 (total respondent) due to non-applicable/non-response.

‡ The national average monthly household income in Malaysian Ringgit (RM) is RM3,686 (US$1 = RM3.08, as of November 6, 2010)

Non-Malay bumiputra was not included in logistic regression due to small number

Model 1 Binary logistic regression for illegal motorcycle street racing racing frequency very often/often versus sometimes/rarely; Hosmer and Lemeshow test, χ^2 ^(8) = 11.228, *P *= 0.189

Model 2 Binary logistic regression for racing and perform stunts versus racing only; Hosmer and Lemeshow test, χ^2 ^(8) = 4.787, *P *= 0.780

Those involved in street racing along with performing dangerous stunts while riding consisted of half of the total participants (50.1%, *n *= 1014). No significant association between duration of racing and stunt riding was found. However, a total of 37.8% of those with duration of racing less than a year reported performing dangerous stunts while riding. In regard to alcohol used while racing, a total of 35.8% (*n *= 724) reported racing under the influence of alcohol. The rates for racing under the influence of alcohol were lower among the Malays (34.8%) compared to Chinese (58.8%) and Indian (40.0%) participants (χ^2 ^= 17.32, *df *= 2, *p *< 0.001). Additionally, participants who were more educated were less likely to race under the influence of alcohol (primary 49.2% *vs*. secondary 36.2% *vs*. university 26.8%; χ^2 ^= 26.70, *df *= 2, *p *< 0.001). Participants who reported that neither parent was strict (52.0%) or only one parent was strict (34.4%) were more likely than participants who reported that both parents were strict (22.9%) to race while under the influence of alcohol (χ^2 ^= 108.50, *df *= 2, *p *< 0.001).

Mean duration of racing was 2.65 (*SD *= 1.77) years, ranged from 2 months to 12 years, with the majority (60.8%) reporting involvement in illegal street racing of between 2 to 4 years. As indicated in Table 1, unemployed participants and those of lower education were more likely to have higher frequency of racing. Family characteristics also significantly influence the frequency of racing.

The mean health protective behaviour score was 4.10 ± 2.1 (possible scores ranged from 0 to 8) and the mode was 4. The scores varied significantly between groups classified by marital status, educational attainment, family household income, maternal and paternal education attainment, and parental strictness. Health protective behaviour scores were significantly higher in married participants (4.67 married *vs*. 4.07 never married, *p *< 0.05), those with higher education attainment (4.23 secondary *vs*. 3.73 primary, *p *< 0.001), high family household income (3.97 < RM1000 *vs*. 4.44 RM2001-3000, *p *< 0.01), high maternal (4.26 tertiary *vs*. 3.79 primary, *p *< 0.001) and paternal (4.39 tertiary *vs*. 3.87 primary, *p *< 0.001) education attainment, and strict parents (4.33 both parents strict *vs*. 3.99 at least one parent strict, *p *< 0.01). No significant differences were found by ethnicity, participants' age and monthly income, locality and parents' marital status.

With regard to health risk behaviours, cigarette smoking was highly prevalent (78.3%), followed by general alcohol use (27.8%) and recreational drug use (18.8%). The mean health risk behaviour score was 2.10 ± 1.0 (possible scores ranged from 0 to 5) and the mode was 2. Only 4.0% and 28.7% reported 0 and 1 risk behaviour, respectively. A total of 24.3% reported between 3 to 5 risk behaviours. There were statistically significant differences in the mean score of health risk behaviour with regard to marital status, participant education attainment, locality, parental marital status, maternal and paternal education attainment, and parental strictness. Health risk behaviour scores were significantly higher in married participants (*p *< 0.015) and those with higher education attainment (*p *< 0.01), urban locality (*p *< 0.001), separated or divorced parents (*p *< 0.001), high maternal (*p *< 0.001) and paternal education attainment (*p *< 0.001), and strict parents (*p *< 0.001).

The mean masculinity score was 15.7 with a standard deviation of 4.0. Possible scores ranged from 0 to 21. The distribution of masculinity scale scores was negatively skewed, indicating that on average study participants scored high on perceived masculinity. The mean score differed significantly by ethnicity and marital status. The Malay participants had highest masculinity score (15.9), followed by Indian (14.6) and Chinese (12.9) participants (*p *< 0.001). Those who were not married had a higher mean masculinity score (15.7) than those who were married (14.6) (*p *< 0.05). No statistically significant differences were observed for age, participant education attainment and income, locality, or other family characteristics variables. Spearman rank order correlations showed that masculinity scores were inversely related to health protective behaviour scores (*r *= -0.056, *n *= 2022, *p *< 0.05) and positively correlated with risky behaviour scores (*r *= 0.059, *n *= 2022, *p *< 0.05). Health protective behaviour scores were inversely related to risky behaviour scores (*r *= -0.10, *n *= 2022, *p *< 0.001).

As shown in Table 1, the result of binary logistic regression analysis for variables associated with illegal motorcycle street racing frequency (Model 1) showed that the variables ethnicity, participant education attainment, occupation, parents' marital status, household income, maternal education attainment and parental strictness, were statistically significant predictors, χ^2 ^= 172.02, *df *= 23, *p *< 0.001. The odds of racing frequency very often/often *vs*. sometimes/rarely increased with health risk behaviour and masculinity scores. The Malays and Chinese had higher odds of racing frequency very often/often *vs*. sometimes/rarely compared to the Indians. The odds of racing frequency very often/often *vs*. sometimes/rarely decreased as participant education attainment and household monthly income increased. Participants with neither or at least one parent who are strict had higher odds of racing frequency very often/often *vs*. sometimes/rarely than those with both parents who are strict.

The binary logistic regression analysis Model 2 showed that only ethnicity, participant education attainment and parental strictness were significantly associated with performing stunt riding while racing, χ^2 ^= 244.49, *df *= 14, *p *< 0.001. The results showed that the Malays and Chinese were more likely to race along with performing stunts while riding compared to the Indians. Participants with neither or at least one parent who are strict were more likely to perform stunt riding while racing than those with both parents who are strict. Participants with higher education attainment had lower odds of involvement in racing along with performing stunts while riding versus racing only. Health-protective, risk behaviour and masculinity scores were also predictive of stunt riding.

## Discussion

Despite the sample being diverse, with a range of socio-economic profiles, the majority were students and those with lower levels of employment. The average monthly income of those who were in employment and the monthly household income were notably lower than the RM3686 national average monthly household income [27]. This indicates that, similar to those involved in car racing [8], most of the illegal motorcycle racers were also from lower socio-economic backgrounds. This study found that a variety of delinquent behaviours exist concomitantly with illegal motorcycle street racing. It is important to note that racing under the influence of alcohol and stunt riding were highly prevalent. It is also worth noting the worrying fact that **s**lightly over one-third of those reporting duration of racing less than a year reported performing dangerous stunts while riding. In our recent in-depth interview study (Wong LP: In-depth understanding of health risk behaviors and needs of illegal motorcycle racers in Malaysia, submitted), illegal motorcycle racers performed dangerous stunts while riding to gain respect and honour from peers and the street racing community for being brave and tough. Many also reasoned that boredom and lack of recreational activities for leisure participation contributed to their involvement in street racing, while a minority viewed street racing as an effective means to reduce stress (Wong LP: In-depth understanding of health risk behaviors and needs of illegal motorcycle racers in Malaysia, submitted).

As students represent a large majority of illegal street racers, and there were a number racers aged under 15 years, early intervention services including education and prevention programs for at-risk adolescent and their families, as well as counseling and mentoring at school level, may be beneficial for this group. Although motorcycle street racing is known to be predominant among the Malays, this study found a considerable number of other ethnic groups, such as the Chinese and Indians, also participate in this high risk activity. As such, culturally competent prevention or intervention program for our ethnically diverse community is warranted. For example, previous research has suggested that street outreach programs coordinated by trained peer leaders are effective in reducing risky behaviours [28,29].

The health protective behaviour score of 4.1 out of 8 indicates the need for initiatives to enhance health promotion, illness prevention, and preventive health behaviours among the illegal street racers. In terms of health risk behaviours, the finding is consistent with problem behaviour theory where problem behaviours are rarely an isolated behaviour, and often embedded in various other forms of negative behaviours [11,12]. For example, adolescents who frequently use cigarettes were also more likely to use alcohol and marijuana [30]. The current study found that cigarette smoking was most prevalent among the motorcycle racers, followed by alcohol drinking and drug use. It has been reported that the consequences of these risk behaviours may influence multiple domains of adolescents' lives. It may interfere with motivation and thinking processes, and increase the risk of serious medical complications, accidental death, and other violent crimes [4]. The coexistence of smoking, alcohol drinking and recreational drug use among the illegal motorcycle racers appear to warrant intervention tailored for youth population to effectively prevent or discontinue these health risk behaviours.

In logistic regression analyses, variables such as low education attainment, low household income, divorced families and households in which parents hold permissive attitudes were associated with higher street racing frequency. Likewise, low educational attainment and households in which parents hold permissive attitudes were more likely to perform dangerous stunts while riding. This indicates that street racers from lower socio-economic groups were particularly vulnerable to motor racing-related risk behaviours, and presumably many other risky behaviours. Therefore, tailoring community interventions to the needs of the lower socio-economic groups may be beneficial. The present study also highlights the magnitude of the influence of parenting style and family structure in motor racing-related risk behaviours. This indicates a clear need for family-centered interventions or parent training for promoting positive parental relationships and authoritative parenting style. In this study, high masculinity and risk behaviour scores were associated with high street racing frequency and likelihood to perform dangerous stunts while riding. Many negative consequences of high masculinity have been found. Masculinity is defined as embracing risk behaviours and opposing positive health behaviours [22], which was supported in this study by a significant inverse correlation between masculinity and health protective behaviour scores. Nevertheless, it should be noted with caution that a Type I error may occur owing to the small but statistically significant correlation coefficient of the association, which may be due to large sample size effect. The findings from this study are consistent with those of earlier studies that showed masculinity as an important construct for health risk behaviours, including unsafe driving habits and illegal street racing [20,25]. Given these associations, interventions recognizing the negative consequences of experimentation and risk behaviours related to street racing as an expression of traditional masculinity should be emphasized.

The study has some potential limitations. The first limitation of the study was the use of snowball sampling and street outreach strategy, which may have resulted in selection bias, and a sample that may not be representative of all segments of population under study. However, it was not possible to assess the extent of any bias given that the population of individuals who engage in illegal motorcycle street racing is unknown. Nevertheless, the study has the advantage of being one of few that have been conducted with such a large sample of illegal motorcycle racers, which may reduce the margin for error, although it may not completely eliminate survey bias. Secondly, information bias may have occurred because data were based on self-report and could not be validated. However, given the sensitivity of the issue investigated, ethical and practical considerations limited the use of other direct assessment methods. Thirdly, the internal consistency (Cronbach's alpha) of some scales (e.g. the health protective and health risk behaviour scales) was less than 0.7. In view of these limitations, the study findings may need to be interpreted with caution.

## Conclusions

The findings from this study have several implications for reaching out to youth known to be associated with illegal motorcycle street racing. The study found illegal motorcycle street racing is associated with a variety of participant, parent and family characteristics. There is a need for increasing emphasis on designing health promotion interventions for low socio-economic individuals as they are particularly vulnerable to becoming involved in illegal street racing. Targeting and educating the lower socio-economic street racers may assist in lowering the rates of risk behaviour and injury due to street racing. Higher level of parental monitoring and control may prevent motorcycle racing-related behaviours among adolescents and youths. The distinctive link between masculinity and street racing indicates that intervention and preventive messages should highlight the negative aspects of masculinity and aimed to change youth's notions of masculinity that often emphasize risk taking. Comprehensive behavioural interventions to simultaneously address the multiple and inter-related risky behaviour exhibited by the illegal racer is warranted. The findings of this study can inform the development of future programs aimed at adolescents and young adults, and may be particularly useful in developing interventions to eradicate illegal motorcycle street racing.

## Competing interests

The authors declare that they have no competing interests.

## Authors' contributions

WLP planned and organized the data collection, carried out data analysis, drafted and revised the manuscript.

## Pre-publication history

The pre-publication history for this paper can be accessed here:

http://www.biomedcentral.com/1471-2458/11/446/prepub
